# Demonstration of multiple quantum interference and Fano resonance realization in far-field from plasmonic nanostructure in Er^3+^-doped tellurite glass

**DOI:** 10.1038/s41598-022-08858-x

**Published:** 2022-03-23

**Authors:** G. Lozano C., O. B. Silva, F. A. Ferri, V. A. G. Rivera, E. Marega

**Affiliations:** 1 Instituto de Física de São Carlos, Universidade de São Paulo, Postal Box 369, São Carlos, SP 13560-970 Brazil; 2 Universidade Estadual do Piauí, Piripiri, PI 64260-000 Brazil; 3 Departamento de Física, Universidade Federal de São Carlos, São Carlos, SP Brazil; 4 Centre d’Optique, Photonique et Laser, Université Laval, Quebec, G1V 0A6 Canada; 5 Facultad de Ciencias Físicas, Universidad Nacional Mayor de San Marcos, Lima, Peru

**Keywords:** Nanophotonics and plasmonics, Optical materials and structures

## Abstract

It is crucial to control the tuning and improve the emission of a quantum emitter at the nanoscale. We report multiple Fano resonances in metallic nanostructures on an Er^3+^-doped tellurite glass. Periodic nanoslits were fabricated with a focused gallium ion beam on a gold thin film deposited on the tellurite glass. Is proposed a coupling function with Fano line-shape form, and the asymmetric parameter *q* for each resonance wavelength in the 515 to 535 nm region was calculated. This asymmetric resonance effect is a consequence of the quantum interaction between the continuum state, generated in the nanostructure, and the Stark splits of the $^2$H$_{11/2}$ state.

## Introduction

The progress in nanophotonics research brings several examples of resonant optical phenomena related to the physics of Fano resonances, with applications in optical switching and sensing, for example^1^. For the practical design of nanophotonic devices, it is important to improve our knowledge about different resonant phenomena. Fano resonances describe strong asymmetries in the autoionization spectra from the quantum interference between two competing transitions^2,3^. This interference configuration is employed in nuclear, atomic, and solid-state physics^4–8^. As well as in photonics and plasmonics to describe single or even double resonance systems^9,10^, taking advantage of the asymmetric line-shape to improve sensor sensitivity^11^ and using their local variations as an intrinsic interferometer^12,13^. Such asymmetric line-shape is results from the interference between a resonant mode and a flat background, whose phase difference produces varieties of line-shapes^14^. In plasmonics, the coupling of radiative and dark modes mimics the atomic electromagnetic-induced transparency^15^.

On the other hand, plasmonic nanostructures can be considered nanocavities with ultra-small mode volumes able to intercede extremely strong self^16–21^ and mutual^22–25^ emitter interactions with large bandwidth and abundant topologies at the deep subwavelength scale. Such nanocavities are also known to be efficient nanoantennas capable of tailoring the excitation^26–28^ and radiation^29–32^ of single emitters, providing large degrees of freedom for system addressing. Based on these superior properties, plasmon-emitter nanosystems exhibit potential applications in testbeds and building blocks for quantum optics and informatics^33,34^. Besides, they are presently the only room-temperature system to achieve a strong coupling regime at the single-emitter level^17,18,21^.

Furthermore, vitreous materials, particularly tellurite glasses containing rare-earth ions (REI), are promising substrates (gain medium) for applications in plasmonics and photonics. These glasses exhibit high linear and nonlinear refractive indices, wide transmission (400 to 5500 nm), good thermal and mechanical stabilities, low cost of production, and large solubility of REI^35–38^. Moreover, noble metal nanoparticles embedded in erbium-doped tellurite glasses can improve the spectroscopic properties for optical applications in telecommunication bands^39^, among other applications^40^. Following this reasoning, the interaction between Er^3+^ and silver nanoparticles acts as a physically realizable damped oscillator on a nanoscale with potential applications for building models for a wide variety of fascinating physical processes in a quantum system, known today as quantum plasmonics^41–43^. The improvement in photoluminescence quantum yield to values comparable with the REI would make plasmonic nanostructures a strong candidate for next-generation optical labels in the fields of optical telecommunication, gas sensing^44^, optical temperature sensing^45^ and imaging^41^.

In this paper, we develop an experimental and phenomenological approach to show multiple quantum interference, including Fano resonances, via micro-transmission measurements in plasmonic nanostructures (periodic array of slits) in a gold thin film on an Er^3+^-doped tellurite glass. In this manner, the proposed experimental results exhibit, for the first time, one of the fundamental properties of Fano resonance between an Er^3+^ and a plasmonic nanostructure.

## Results and discussion

**Figure 1:**
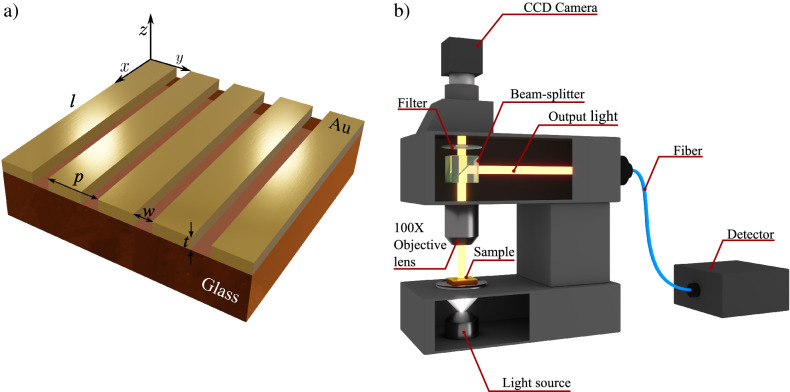
(**a**) Schematics of the grating nanostructures in a gold thin film on tellurite glass as a substrate, where *p*, *w*, *t* and *l* are the period, width, thickness and length, respectively. (**b**) Experimental setup, where a *y*-polarized white light source illuminates the samples from above, and the transmission spectra were recorded in the detector (Ocean Optics USB 2000 spectrometer).

The grating nanostructures were fabricated in 200 nm Au film on tellurite glass substrates using a focused ion beam (see “Methods”), as seen in Fig. 1a. Hybrid systems were labelled as p400, p500, p700 and p900 based on the period values of 400, 500, 700 and 900 nm, respectively. For nanostructures fabricated in the Er^3+^-doped glasses, all labels end with “-Er”. The experimental setup for the optical measurements is illustrated in Fig. 1b.

As reported in a previous study^46^, the density and Er^3+^ ion concentration of the doped glass is 5.11 g cm$^{-3}$ and 1.92 × 10$^{20}$ ions cm$^{-3}$, respectively. Moreover, the refractive index at 532 nm is 2.115 and indicates strong light confinement in small volumes. According to the ion concentration and nanostructure dimensions, approximately 10$^{5}$ Er^3+^ ions are located in the vicinity of each slit. These ions are more likely to interact with the nanostructures. All these characteristics are desirable to analyse the coupling between the emission/excitation of REI with plasmonic nanostructures^41^.

**Figure 2:**
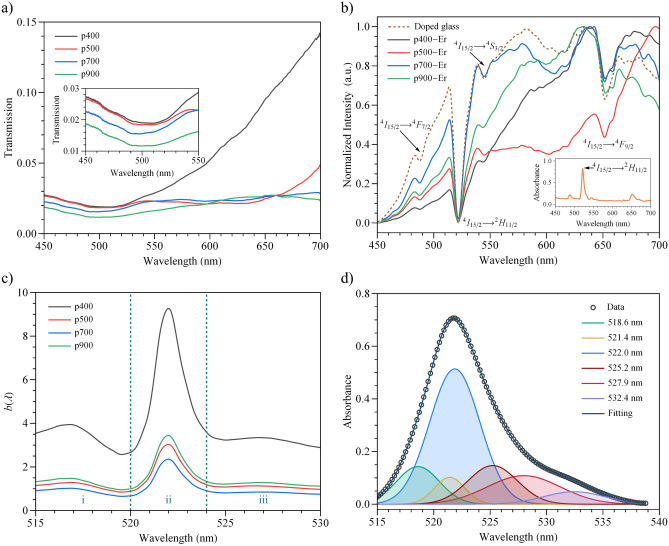
(**a**) Transmission spectra of the nanostructures for the undoped glass. Inset: Transmission in the wavelength range of 450–550 nm. (**b**) Normalized spectra of the transmitted light through the doped glass without (dashed lines) and with nanostructures (solid lines) for different period values showing the characteristics Er^3+^ energy levels. Inset: Absorption spectrum of the doped glass, where the band centred at 522 nm is labelled. (**c**) Coupling function $b(\lambda )$ divided into three regions i, ii and iii. (**d**) Deconvolution of the $^4$I$_{15/2}$ $\rightarrow$ $^2$H$_{11/2}$ absorption band with baseline correction obtained from the doped glass. All measurements were performed in the far-field regimen.

In Fig. 2a, a resonance wavelength can be seen in the range of 500–510 nm with broadband from 450 to 550 nm for all nanostructures in the undoped tellurite glass. In addition, the transmission in this region slightly decreases with the increment of the period. This last is because while increasing the period, losses for reflectance at short wavelengths are expected, as reported in Ref.^47^. Besides, a notorious peak can be observed in the region greater than 700 nm due to the extraordinary optical transmission (EOT) of light, and it goes beyond the wavelength range of study. Figure 2b illustrates the normalized transmission through the doped glass without (dashed lines) and with nanostructures (solid lines), showing the characteristic Er^3+^ absorption bands as well as in the absorption spectrum of the undoped glass plotted in the inset. Here, the main absorption band is centred at 522 nm, which corresponds to the $^4$I$_{15/2}$ $\rightarrow$ $^2$H$_{11/2}$ transition, and its high value is characteristic in tellurite glasses^48^. The other observed bands are $^4$I$_{15/2}$ $\rightarrow$ $^4$F$_{7/2}$, $^4$I$_{15/2}$ $\rightarrow$ $^4$S$_{3/2}$ and $^4$I$_{15/2}$ $\rightarrow$ $^4$F$_{9/2}$ centred at 488, 544 and 652 nm, respectively. The purpose of the normalization of the transmitted intensity spectra is to observe the changes in the line-shape based on the spectrum through the doped glass without nanostructures. In this frame, a quantum interference (QI) between the discrete absorption peaks of the Er^3+^ and the continuum absorption from the plasmonic nanostructure is expected. Nevertheless, in Fig. 2b, it is impossible to observe such QI directly from the experimental setup. Therefore, it is necessary to define the coupling mechanisms in this hybrid system, which should display a Fano resonance, i.e., a coupling function to describe the line-shape which is unable to detect in the far-field measurements. To obtain such function, we define $G(\lambda )$ as the transmitted intensity line-shape of the nanostructure in metallic film on the glass, $H(\lambda )$ as the transmitted intensity line-shape of the glass doped with Er^3+^, and $I(\lambda )$ as the transmitted intensity line-shape of the nanostructure in metallic film on the glass doped with Er^3+^. Hence, we defined a coupling function $b(\lambda )$ such that:

1 $$\begin{aligned} I(\lambda ) = b(\lambda ) G(\lambda ) H(\lambda ) \end{aligned}$$

The coupling function $b(\lambda )$ gives information about the kind of QI of the hybrid system. The obtained coupling functions are plotted in Fig. 2c and exhibits multiple asymmetric profiles, which puts in evidence a resonant QI between the excitation of the $^2$H$_{11/2}$ discrete state (i.e., their manifold levels are to the Stark effect) and the continuum attributed to the nanostructure. It is important to mention that a strong coupling (symmetric or not) only occurs between the ions near the nanostructure. Otherwise, we will only have an EOT of the ions emission far from the slits. In addition, the coupling function cannot be observed elsewhere because the $^4$I$_{15/2}$ $\rightarrow$ $^2$H$_{11/2}$ transition is hypersensitive and shows a strong oscillator strength^48^ compared with the other bands in our region of interest, and beyond 550 nm is only observed EOT ($^4$I$_{15/2}$ $\rightarrow$ $^4$F$_{9/2}$ transition), which does not generate QI in this system. Further, the $^2$H$_{11/2}$ energy level may be splitting into a maximum of $(2J+1)/2=6$ energy levels due to the Stark effect^49^. These levels have distinct centre peaks and bandwidths depending on the glass host^50^. The observation of these multiple asymmetric line-shapes can be a response of these energy levels interfering with the plasmon and reasonably assigned as a multiple Fano resonance (see Fig. 2c). Such energies are obtained by deconvolving the $^4$I$_{15/2}$ $\rightarrow$ $^2$H$_{11/2}$ band of the doped glass. For this purpose, the *FityK* software was used based on gaussian functions and the Levenberg–Marquardt algorithm in the wavelength range of 515–530 nm as illustrated in Fig. 2d, where six deconvolved bands were obtained (R$^2$ > 0.99) and shall be discussed later.

**Figure 3:**
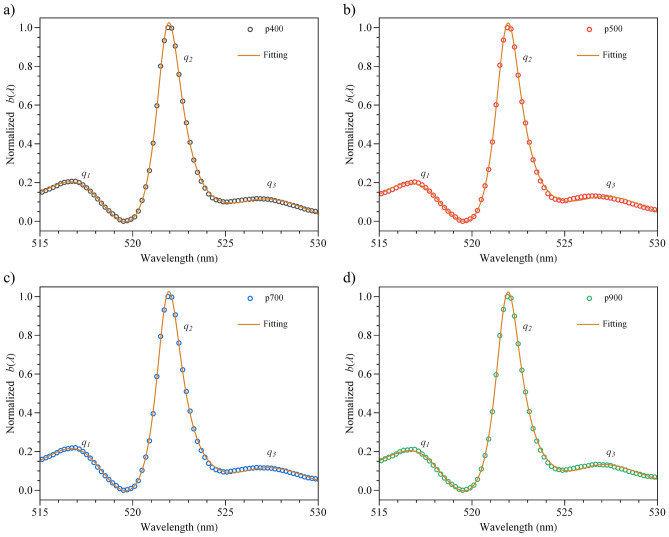
Fitting of the normalized $b(\lambda )$ parameter for all nanostructures with an adjusted R$^2$ > 0.99. Each asymmetric shape is associated with a $q_j$ value.

**Figure 4:**
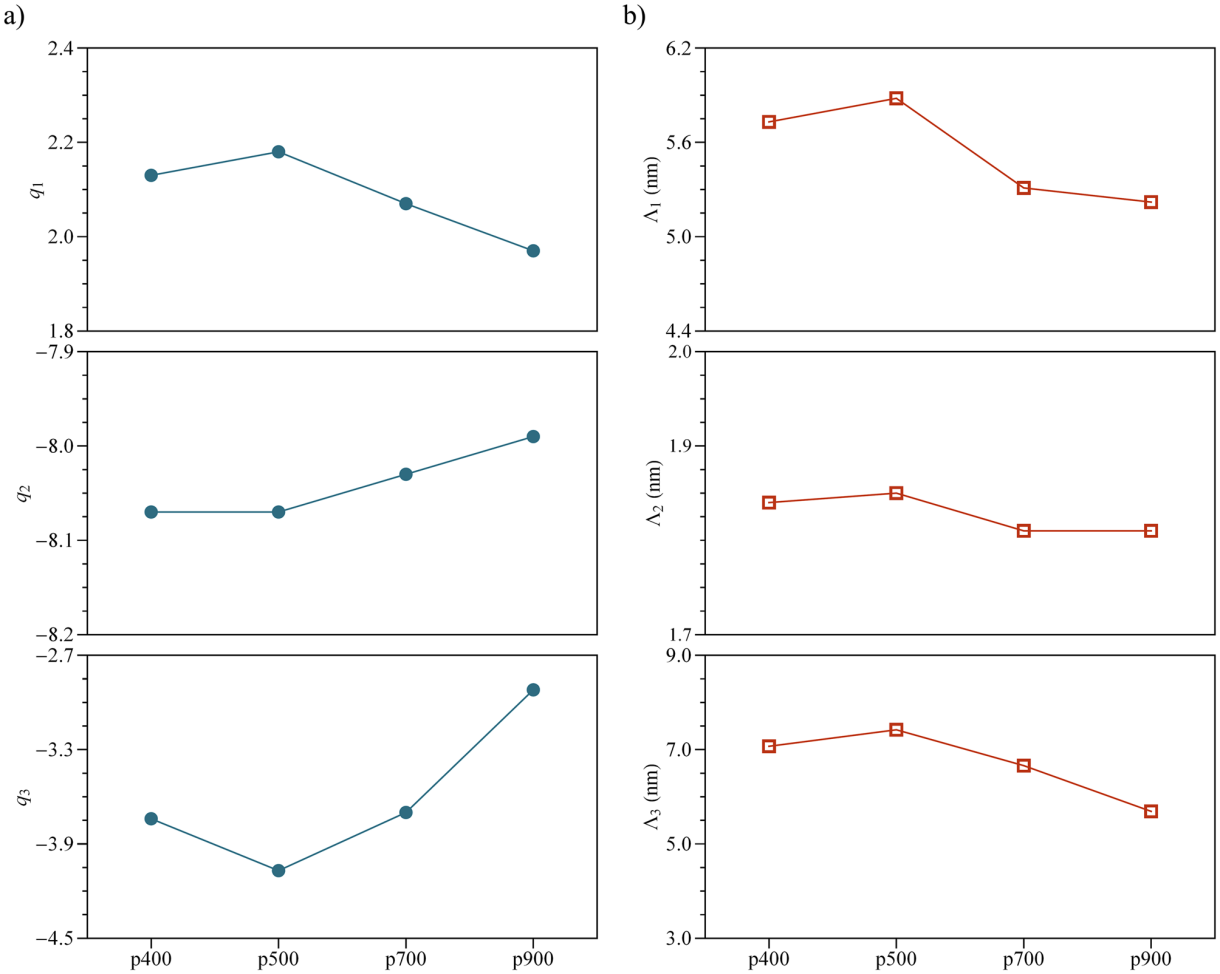
(**a**) *q* parameter (dimensionless) calculated for each period value. (**b**) The resonance width $\Lambda$ (in nm) shows a slight decrement with the increment of the period.

As established above, the results show a multiple Fano resonance effect in this hybrid system. In Fig. 2c, three regions were considered, where the asymmetric line-shapes are well defined, and each one is assumed as a Fano line-shape function^10^. Therefore, to corroborate this hypothesis, a fitting for each $b(\lambda )$ coupling function was performed by using the following expression:

2 $$\begin{aligned} b(\omega ) = b_0 + \sum _j A_j \frac{\left( q_j \Gamma _j / 2 + (\omega -\omega _j)\right) ^2}{(q_j \Gamma _j / 2)^2+(\omega -\omega _j)^2} \end{aligned}$$

where $q_j$ is the Fano parameter, $\omega _j$ is the resonance frequency, $\Gamma _j$ is the resonance width and $A_j$ is the weight ($j = 1,2,3$). Figure 3 illustrates the fitting for each normalized curve in the wavelength range of 515–530 nm (R$^2$ > 0.99). The choice of three terms in the sum is a good approximation since some split energy levels contribute to a lower degree. In Fig. 4a,b, the parameters $q_j$ and $\Gamma _j$ are displayed, respectively, where the following changes $\omega _j$ $\rightarrow$ $\lambda _j$ and $\Gamma _j$ $\rightarrow$ $\Lambda _j$ were performed to express these quantities in the units of nm. $\lambda _j$ is the corresponding wavelength at the frequency $\omega _j$, and we define $\Lambda _j$ as $2\pi c \left( \frac{1}{\omega _j - \Gamma _j/2}-\frac{1}{\omega _j +\Gamma _j/2}\right)$, where *c* is the speed of light. For all nanostructures, the resonance wavelengths are approximately centred at 518, 522 and 528 nm where, at 522 nm, the coupling function exhibits its maximum, and the $q_2$ parameters are approximately 8, indicating a subtle symmetry line-shape^10^. Conversely, the values of $\vert q_1 \vert$ and $\vert q_3 \vert$ are lower than $\vert q_2 \vert$ and indicate a stronger asymmetric resonance. Additionally, the resonance wavelengths match with three deconvolved wavelengths ($\approx$ 518.6, 522.0 and 527.9 nm), as seen in Fig. 2d. To explain the difference between the absolute values of the $q_j$ parameter, we consider that the probability of one of the $^2$H$_{11/2}$ Stark splits to be excited is greater since the deconvolved band centred at 522.0 nm represents $\approx$ 48.7% of the total area (see Fig. 2d). The contribution of the 521.4 nm peak should also be considered since its area represents 5.8% and therefore enhanced the amplitude to a greater extent than interfere. Moreover, a multiple QI between the six Stark levels observed in Fig. 2d may neglect the coupling with the continuum. The 518.6 and 527.9 nm deconvolved bands represent 11.5 and 14.9% of the total area. However, their influence on the resonance is given by the kind of interference, constructive or destructive (stronger asymmetric resonance), between these split levels and the continuum. Regarding the Er^3+^ ions near the metallic film, no Fano resonance is detected in the states below $^2$H$_{11/2}$ because the oscillator strengths of these levels are less intense in tellurite-germanate glasses^46^ and, therefore, could achieve (or not) a weak coupling. No remarkable shifts in each coupling function are observed as the resonance wavelengths maintain approximately their same values. It is important to remark that, for the *q* parameter (see Fig. 4a), a trend can be noted starting from the computed values for p500, where $q_1$, slightly decreases while $q_2$ and $q_3$ slightly increase. Similarly, a trend is observed in the resonance width from Fig. 4b, where all $\Lambda _j$ values decrease starting from p500. The period of this nanostructure is near the calculated resonance wavelengths (518, 522 and 528 nm). Thus, this hybrid system is period-sensitive.

**Figure 5:**
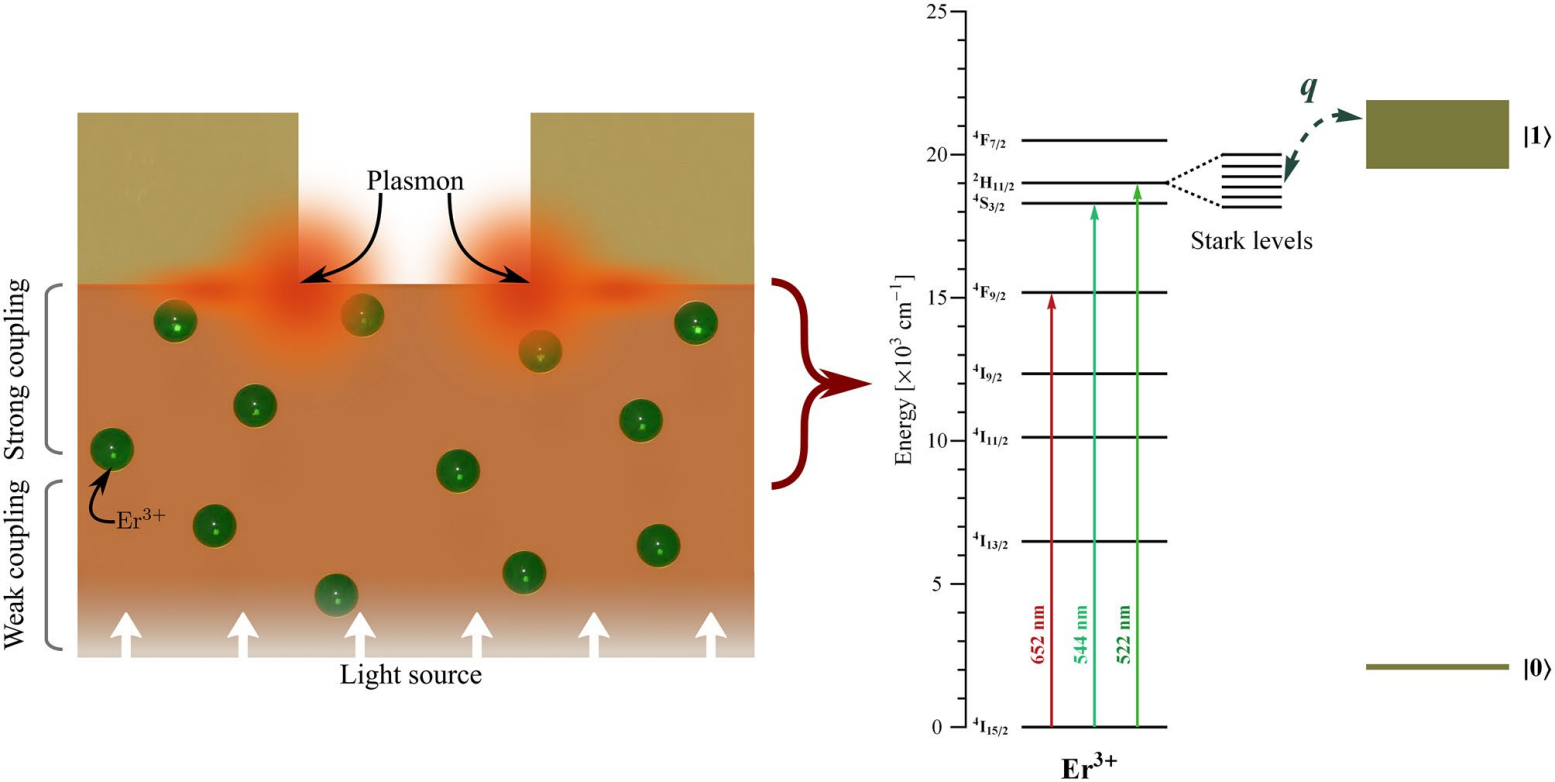
Representation of the plasmonic nanostructure and Er^3+^ coupling and energy level diagram of the Er^3+^ and the plasmon (denoted as $\left| 0\right\rangle$ $\rightarrow$ $\left| 1\right\rangle$) where the Fano resonance, represented by *q*, is observed. The $^2$H$_{11/2}$ energy level is split into 6 Stark levels. The Er^3+^ ions are represented as green spheres.

Figure 5 summarizes the coupling process between the Er^3+^ ions and the nanostructure: We assume a strong coupling between the nearest Er^3+^ ions and the nanoslits. This kind of coupling generates a photon-plasmon conversion effect, where the light travels through the glass–gold interface as plasmon and goes out through the slit to be detected as photons. From the literature, a strong coupling is obtained when the Er^3+^ ions are near the nanostructure, which is evidenced by the enhancement of the emission or excitation processes due to the increment of the local field^51–53^. The $^2$H$_{11/2}$ state splits into six manifold levels, and each one experiences a constructive/destructive interference with the continuum state of the plasmon and generates QI, which is denoted as $\left| 0\right\rangle$ $\rightarrow$ $\left| 1\right\rangle$, where $\left| 0\right\rangle$ represents the plasmon ground state and $\left| 1\right\rangle$ the resonance modes of the nanostructure including the plasmon generated at the glass-gold interface. The other energy levels do not interfere because the farthest Er^3+^ ions in the glass–gold interface weakly interact with the nanostructure. We only observe EOT of the $^4$I$_{15/2}$ $\rightarrow$ $^4$F$_{7/2}$, $^4$I$_{15/2}$ $\rightarrow$ $^4$S$_{3/2}$ and $^4$I$_{15/2}$ $\rightarrow$ $^4$F$_{9/2}$ transitions, as shown in Fig. 2b. Some of these Stark levels exhibit a notorious Fano resonance effect (see Fig. 2c), which were quantified with the *q* and $\Lambda$ parameters obtained from the proposed coupling function $b(\lambda )$.

In summary, multiple Fano resonance in the far-field was observed, for the first time, in a system of periodic gold nanostructures in Er^3+^-doped tellurite glass. In the proposed $b(\lambda )$ coupling function, the magnitude of the coupling/resonance is not given by the amplitude but by the asymmetry profile since $b(\lambda )$ has the form of a Fano asymmetric line-shape. This hybrid system is nanoslit period sensitive since it was found that a trend with the values of *q* and start where the period matches with the calculated resonance wavelengths. These results are crucial for a better understanding of the coupling between quantum emitters, as a REI, and a plasmonic nanostructure. Finally, the results also allow us to face the fabrication of new devices that can control the tuning and improve the emission of a quantum emitter at the nanoscale.

## Methods

### Sample fabrication

Two tellurite glass substrates with nominal composition (75$-x$) TeO$_2$–5GeO$_2$–10Na$_2$O–10Nb$_2$O$_5$–*x*Er$_2$O$_3$ (in mol%), where $x = 0$ and 1, was prepared by the conventional melt—quenching technique: the samples were melted at 800 $^\circ$C for 30 min and then annealed at 300 $^\circ$C for 240 min and slowly cooled down to room temperature (rates of 1 $^\circ$C/min). The glasses were cut and polished to obtain $\approx$ 2 mm in thickness. Afterwards, a gold thin film, with 200 nm in thickness, was deposited on both glasses by conventional sputtering. Metallic gratings with narrow slits were fabricated for both doped and undoped glasses with a focused ion beam (FIB) Dual Beam FEI Quanta 3D 200i (Ga$^+$ ions, 30 keV) with the following dimensions: *t* = 200 nm, *w* = 50 nm, *l* = 2000 nm and *p* = 400, 500, 700 and 900 nm as illustrated in Fig. 1a. Then, the nanostructures were labelled based on the period value as p400, p500, p700 and p900. At the end of each label, “-Er” was added to indicate that the fabricated nanostructure is on the Er^3+^-doped substrate.

### Optical measurements

The intensity of the transmitted light was obtained by integrating the signal in the entire region of interest in an Ocean Optics USB 2000 spectrometer (450 to 700 nm) and subtracting the background caused by electronic noise. The light source is a tungsten lamp, the microscope is an Olympus BX61-W1, and the features of the hybrid system and experimental setup can be seen in Fig. 1a,b, respectively.
